# The uses of fig (*Ficus*) by five ethnic minority communities in Southern Shan State, Myanmar

**DOI:** 10.1186/s13002-020-00406-z

**Published:** 2020-09-17

**Authors:** Aye Mya Mon, Yinxian Shi, Xuefei Yang, Pyae Phyo Hein, Thaung Naing Oo, Cory W. Whitney, Yongping Yang

**Affiliations:** 1 Key Laboratory of Economic Plants and Biotechnology, Kunming Institute of Botany, Chinese Academy of Sciences, Kunming, 650201 China; 2 University of Chinese Academy of Sciences, Beijing, 100049 China; 3 Southeast Asia Biodiversity Research Institute, Chinese Academy of Sciences, Nay Pyi Taw, 05282 Myanmar; 4 Forest Department, Ministry of Environmental Conservation and Forestry, Nay Pyi Taw, 05282 Myanmar; 5 Institute of Crop Sciences and Resource Conservation (INRES), Horticulture Institute, University of Bonn, Auf Dem Huegel 6, 53121 Bonn, Germany; 6 Center for Development Research (ZEF), University of Bonn, Genscherallee 3, 53113 Bonn, Germany; 7 Plant Germplasm and Genomics Center, The Germplasm Bank of Wild Species, Kunming Institute of Botany, Chinese Academy of Sciences, Kunming, 650201 China

**Keywords:** *Ficus*, Ethnobotany, Traditional knowledge, Sustainable development, Medicine, Conservation, Myanmar

## Abstract

**Background:**

Most regions of Myanmar fall within the Indo-Burma Biodiversity Hotspot and are threatened with biodiversity loss. Development of a comprehensive framework for sustainable development is crucial. Figs are ecological keystone species within these regions and are also important for traditional spiritual food and health uses, which often have accompanying conservation practices. The traditional use and management of figs may offer clues to help guide the development of national policies for sustainable development. In this study, we showcase the rich ethnobotanical knowledge as well as the variety of collection and conservation practices of figs among five ethnic groups in Southern Shan State.

**Methods:**

We performed both key informant and semi-structured interviews with 114 informants from five ethnic groups. Their uses for figs were categorized according to local practices and recipes. Informants were asked about trends in conservation status over the past 10 years and any conservation-related customs and practices. Data were analyzed quantitatively with common quantitative ethnobotany indices, the use report (UR) and use value (UV).

**Results:**

Informants reported the uses of eight fig species (*Ficus auricularta*, *F. concinna*, *F. geniculata*, *F. hispida*, *F. racemosa*, *F. religiosa*, *F. semicordata*, and *F. virens*). *F. geniculata* and *F. virens* were most useful (UR = 228) and were used by all five ethnic groups, corresponding to a high use value (UV = 2). Treatments for 16 diseases were reported from seven species. Household consumption, economic and sacred uses were accompanied by sustainable practices of harvest and protection. Traditional taboos, *in situ* and *ex situ* conservation were common especially for highly demanded species (*F. geniculata* and *F. virens*) and the sacred fig *F. religiosa.*

**Conclusion:**

Findings suggest that figs are useful for food (all informants) and medicine (13.16% of the informants) in the study area. Traditional taboos, *in situ* and *ex situ* conservation practices help to maintain sustainable utilization of locally important figs. This is an early contribution to the traditional knowledge of edible figs. Although similar uses have been reported in neighboring countries for seven of the fig species, the ethnobotanical use of *F. concinna* is novel.

## Background

A large part of Myanmar is located within the Indo-Burma Biodiversity Hotspot, considered to be one of the most biologically important regions and most threatened reservoirs of flora and fauna on the planet [1]. Myanmar seeks to create a comprehensive framework for sustainable development to preserve natural ecosystems, which are considered to be essential in ensuring Myanmar’s development goals for both present and future generations. The long-standing use of figs and traditional conservation practices may yield important knowledge to inform Myanmar’s National Biodiversity Strategy and Action Plan (2015–2020) [2] and Sustainable Development Plans (2018–2030) [3]. Goal five of Myanmar’s Sustainable Development Plan (2018–2030) focuses on the legal, institutional, and policy frameworks to enforce protection and management of ecosystems and to strengthen conservation efforts. The plan also seeks to restore and safeguard ecosystems that provide essential services to ethnic and local communities and the poor and to provide other cultural values. Myanmar has a strong Buddhist culture with practices that include the conservation of figs. For example, the traditional watering festival to the sacred fig tree is held every full moon day of the Kasone month (the second month of the Myanmar lunar calendar, equivalent to the month of May in the Gregorian calendar) in remembrance of the Buddha’s enlightenment under the sacred Bodhi tree 25 centuries earlier [4].

Figs (genus *Ficus* L.; family Moraceae) constitute one of the largest genera of angiosperms with more than 800 species [5] of moderate woody plants or trees, epiphytes, and shrubs. They constitute an important part of the biodiversity in many tropical areas of the world including Indo-Australasia, Neotropical, and Afrotropical regions [6]. Figs are used by humans for food, health, and other functional and cultural purposes throughout their distribution [7]. Notable species of the genus are the sacred fig (*Ficus religiosa* L.), sacred to Hindus and Buddhists and others throughout the world [8]; the sycamore fig or the fig-mulberry (*Ficus sycomorus* L.), also known as the “tree of life” in Egypt [7]; the Indian rubber tree (*Ficus elastica* Roxb. ex Hornem.), one of the few potential crops for producing natural rubber [9]; cluster fig (*Ficus racemosa* L.), considered sacred to the god Dattaguru in India [10]; banyan tree (*Ficus benghalensis* L.), sacred to Hindus and Buddhists; common fig (*Ficus carica* L.), the most popular species that has been cultivated for food and medicine for over 11,000 years [11]; and roxburgh fig (*Ficus auriculata* L.) and white fig (*Ficus virens* Aiton), which are the most commonly consumed species in tropical regions (mainly young leaves and leaf buds) [12].

There are currently 95 botanical records of fig species in Myanmar [13]. At least 20 of these species are considered important ingredients in traditional medicine [14–17], among other uses. Traditional knowledge on fig use is often transmitted orally from generation to generation and is an integral part of many local people’s cultural identity. Some uses are published in ancient medicinal encyclopedias compiled by monks who tend to be the holders and transmitters of traditional religious knowledge [14, 16, 17]. However, formal records and studies on the traditional uses of figs among Myanmar’s diverse ethnic groups are limited, and only a few formal medicinal or nutritional studies have been performed to describe the medicinal plants or to verify reported uses [18, 19]. We hypothesized that these traditional uses often have accompanying conservation practices which may offer clues to help guide Myanmar in generating policy for sustainable development.

The field of ethnobotany can help in defining and strengthening the preservation of native biodiversity. The field offers opportunities for the collection and recording of traditional knowledge and the preservation of the relevant species [20]. Through the current study, we aimed to investigate the ethnobotanical knowledge of figs as well as collection and conservation practices. We offer a case study of knowledge holders from five ethnic groups in the Southern Shan State of Myanmar. Our objective was to document the use and conservation of important figs. We sought to explain (1) the diversity of figs that are of cultural relevance in the study area (Southern Shan State), (2) differences in the diversity of uses and species among the local ethnic groups in four townships of study area, and (3) conservation practices on figs in the study area. The work contributes to the body of knowledge on sustainable use and conservation of figs to support the Myanmar government’s strategic plan for biodiversity conservation [2]. The collection and publication of this knowledge offers recognition for ethnic minorities and indigenous people as experts and as owners of important traditional knowledge. It gives credit for their contributions to the preservation of this knowledge and conservation of biodiversity in Myanmar.

## Methods

### Study area and sample sites (Fig. 1)

Shan State is located on the Shan plateau in the central eastern part of Myanmar (19° 17′ N and 24° 13′ N and 96° 10′ E and 101° 11′ E). It covers 155,800 km^2^, accounting for almost a quarter (23.2%) of the total area of Myanmar. The plateau has an average elevation of 900 m above sea level. Average annual precipitation is between 1900 and 2000 mm [21]. The weather is divided into three seasons. The winter season lasts from November to February; the summer season is from March to June and ends in a rainy season between July and October. Annual mean temperatures range from 12 to 25 °C [22].

**Fig. 1:**
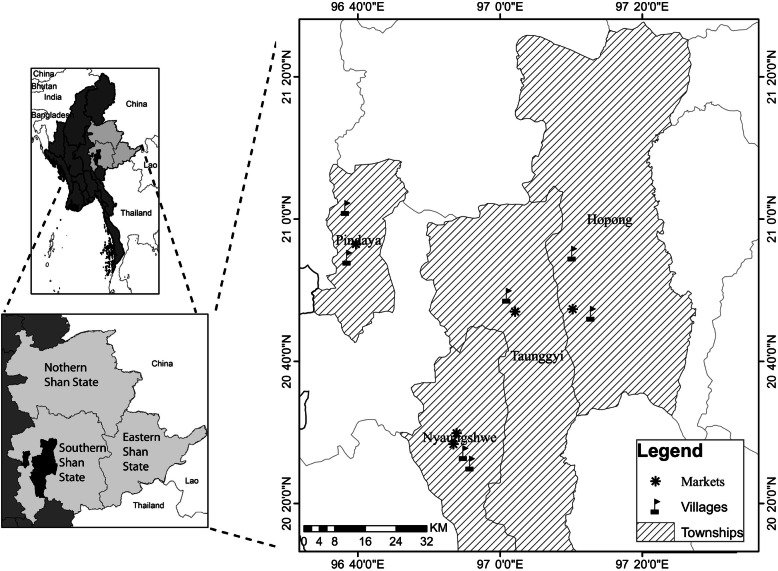
Study area including five markets, five monasteries, and seven villages in Pindaya Township, Nyaung Shwe Township, Hopong Township, and Taunggyi Townships of Southern Shan State, Myanmar

The state is named after the Shan people who make up half the population of the state. Shan State is divided into Northern Shan State, Southern Shan State, and Eastern Shan State [23]; most areas are highly restricted to travel even for local residents and are out of the political control of the central government [24]. It is bordered by China to the north, Laos to the east, and Thailand to the south. The region is home to 33 of the 135 ethnic groups in Myanmar. It is the second most diverse ethnic area after Chin State [24]. The language and culture of the state is similar to that of the Thai neighbors to the east [24]. The state population is 5.8 million, most are Buddhists (81.7 %), followed by Christians (9.8%), Animists (6.6%), Muslims (1%), Hindus (0.1%), and Atheists and others (> 1%) [25]. Local livelihoods are based on paddy rice cultivation in small lowland basins and shifting cultivation of vegetables on the surrounding hillsides [26]. These shifting cultivation practices are giving way to permanent cultivation of cash crops such as tea, orange, and pear. Despite these changes, the wild harvest of subsistence resources from forests is still common and plays an important role in the daily lives of local people. Related local traditional knowledge about nature and plants is important to local people and has been passed down for many generations, largely as part of the local spiritual practices [24].

We chose to work in Southern Shan State as it is the most accessible of the regions of Shan State and hosts 15 of the 95 figs in the Myanmar plant checklist [13]. In order to explore the use of figs in this region, we selected seven villages based on secondary information about the prevalence of fig use and information from fig collectors and sellers at local markets. Informants were identified through snowball sampling [27], this was extremely useful for identifying local people with knowledge about figs, particularly healers and monks who could tell us about medicinal uses.

We conducted surveys in Pindaya Township, Nyaung Shwe Township, Hopong Township, and Taunggyi Township  (Fig. 1). Pindaya Township is a Danu self-administrative area, and most of the local inhabitants belong to the Danu ethnic group [24]. The Danu ethnic group makes up about 4.4% of the 5.8 million people in Shan State. They speak a dialect of Burmese [28]. Their traditional food and medicinal customs include the use of many plants, grown around their homes and collected from forests [29]. The Danu cultivate tea on the hill sides, together with coffee, orange, avocado, cabbage, and mustard crops, as their main source of livelihood.

Inhabitants of the Nyaung Shwe Township are mainly from the Intha ethnic group, who occupy Inle Lake (second largest lake in Myanmar) and around Inle lake with approximately 1.2% of the Shan State population [30]. They speak a Burmese Shan dialect [30] and are famous for their traditional leg-rowing technique [31] and traditional wood and bamboo stilt houses on the water. Their livelihood activities are cultivating tomato and other minor crops on the masses of floating roots and soil, together with fish farms.

Hopong Township is in the Pa-O self-administrative area, with 56% Pa-O ethnic people [32]. They speak their own language and make their livelihoods by cultivating “tha-nat-phet” (*Cordia myxa*), tea, rice, corn, potato, pigeon pea, turmeric, and other garden crops. They rely on indigenous medicine made with medicinal plants.

In Taunggyi Township, most of the informants who we interviewed belonged to the Shan and Bamar ethnic majority of Myanmar. The Shan majority group makes up 35.23% of Shan State’s population. They have mainly settled in valleys and river basins rather than in the mountains. Linguistically, Shan people speak a Tai language and have much in common with Tai [24]. Shan people in most of the armed conflict area have limited access to health care services [33] and largely rely on healers and shamans for medicine. Shan people cultivate rice and vegetables including soybeans, garlic, and corn. The Bamar majority ethnic group occupies about 68% of the total population in Myanmar but only 11.44% in Shan State. They are the main ethnic group of Myanmar and speak Burmese language. The Bamar informants mostly rely on selling vegetables or work as government staff, transferred from other parts of Myanmar.

In our interviews, Shan and Bamar informants were often the ones engaged in selling figs as part of their work in the township markets. All the ethnic groups had knowledge on the use and availability of figs for food, medicine, construction, fencing, as well as for fuel.

### Ethnobotanical data collection

We selected the potential study sites during preliminary field surveys and visits to local vegetable markets in December 2016. We conducted formal surveys with a total of 114 informants from January to February in 2018 and followed up with supplementary field surveys in December 2018 with groups of key informants. The supplementary field survey was conducted to confirm some unclear information and to collect additional voucher specimens for those species without complete information (for example, voucher specimens without fruit). The first meetings were organized with the village leaders, elders, monks, and individual informants, and the objectives of the research were explained. Prior informed consent and approval were received before we started each interview. We conducted fieldwork at three main locations in each township:

In the market, we interviewed 32 sellers about popular and marketable figs at five markets in four townships.

In five Buddhist monasteries, we interviewed six monks, three of them were medicinal experts, and one nun about traditional Buddhist medicinal uses of figs. We also interviewed four members of women’s groups and volunteer groups serving general duties at monasteries.

We interviewed 70 informants in seven villages on the use of figs. Interviews were in villagers’ homes, farming plots, and wild collection sites. We also visited the homes of medicinal experts who were known to be particularly knowledgeable about medicinal plants.

At the beginning of each interview, we asked informants whether they use any figs for dietary purpose. After discussing these, we shared fresh specimens and photographs of those that are commonly used in the area according to the Myanmar plant checklist [13] and nearby areas with similar demographic conditions [12]. We followed ethnobotany theory and methods related to plant species uses [34, 35] and related socioeconomic and conservation practices [36]. To better understand uses, we asked open-ended questions about traditional modes of consumption, medicinal uses, local names and folk names, collection season, and the status of the wild population. For the conservation status, we asked informants to tell us about the availability of the species in the present compared to the past 10 years and their current conservation status. Interviews were carried out in the Burmese language, common to all five ethnic groups, with the assistance of local guides who also provided translation where necessary. We identified the folk names for each plant species and variety. All informants were requested to pronounce the folk names of each species of which we made a voice recording. When there were different pronunciations for one species in one ethnic group, we confirmed it again with key informants. We used the descriptions and pronunciations to compare folk classification.

### Voucher specimen collection

To ensure correct species identification, we collected three or more sets of voucher specimens for each species. Voucher specimens were collected in nearby forests through transect walks [37] with the informants and in the villagers’ home compound if the plant was cultivated. Collection was performed as part of the interview to verify the species and to help in identifying other figs collected and consumed in the area but not necessarily sold in the market. Each voucher specimen was assigned a specific code and GPS location. Photos of fresh and dry specimens were recorded and verified with plant taxonomists at Queen Sirikit Botanic Garden in Thailand as well as the Xishuangbanna Tropical Botanical Garden and Kunming Institute of Botany in China. Voucher specimens were deposited at the herbarium of Kunming Institute of Botany (KUN) in China and the herbarium of Forest Research Institute (RAF) in Myanmar. Accepted taxonomic names were verified with the “World Flora Online” database [38].

### Quantitative assessment

We followed quantitative ethnobotany approaches to allow for assessment of the qualitative data [34, 39]. Categorization is a critical step in quantitative ethnobotany. We generated four categories of fig uses into (1.) food, (2.) acute disease, (3.) chronic disease, and (4.) animal feed (Table 1). We also generated a set of subcategories of uses for food into vegetable and fruits following the process proposed by Bhatia et al. [40]. Vegetable uses were divided into five subcategories of use for young leaves and leaf buds and four subcategories for ripe or unripe fruits following by the local uses and recipes. We also assessed every use report of a health treatment into ten subcategories for acute diseases and six subcategories for chronic diseases. Animal feeding was also divided into two subcategories. Conservation practices for the fig species were also counted (but not categorized).

**Table 1:** Types of uses included in the categorization of fig uses as food, acute diseases, chronic diseases, and animal feed

Use category	Type of uses
Food	
Vegetables	Cooked, fried, eaten raw, salad, reserved
Fruits	Eaten raw, processed, preserved, beverage
Acute diseases	Constipation, cuts and wounds, diarrhea, excessive sweating, herpes, indigestion, irregular mensuration, leucorrhea (vaginal discharge), longevity, postpartum supplement, snake bites, ulcer
Chronic diseases	Diabetes, fever, heart disease, hemorrhoid, hypertension, pulmonary disease, urine disease
Animal feed	Feeding cattle, feeding pigs

We used the Use Report (UR) calculation as the basis for quantitative assessments. A UR is counted when an informant reports the use of a species within a specified use category [41]. For example, roxburgh fig was used as a cooked vegetable by two informants, raw fruit by four informants, and as a heart disease treatment by one informant, giving it a total UR of 7.

We used the Use Value index (UV) to demonstrate the relative importance of a species using the formula *UV=U/N*, where U is the number of citations per species and N is the number of informants [42]. High UV for a plant implies that the plant is useful and a low score indicates that the plant is of negligible importance.

All analyses were performed in R programming language [42] using the ethnobotanyR package [43]. The inter-relationships of general categories between data in resulting matrices are displayed using chord diagrams generated with the ethnobotanyR package. Data on uses for food and medicine and related conservation practices were summarized into an alluvial diagram using the ggalluvial package [44].

## Results

Nearly all informants (99.12%) were native to the place where they were interviewed. The ages of informants varied from 15 to 80 years (Table 1). Out of five ethnic groups, Pa-O represented over half of all informants (they did the most collecting and warranted a heavier research effort), whereas Bamar represented the fewest respondents (mainly market stall managers) (Table 1). In this study, informants mentioned a total of eight species. The species names and voucher specimen numbers for each species are shown in Table 2.

**Table 2:** Demographic information of the 114 informants from five ethnic groups in Southern Shan State, Myanmar

Role	Ethnic belonging					Gender		Age			Total
	Danu	Intha	Pa-O	Shan	Bamar	Female	Male	15–40	41–65	66–90	
Vegetable seller	1	4	19	4	4	29	3	18	13	1	32
Religious person and healer	2	2	4	-	-	1	7	1	4	3	8
Informed consumer (farm cultivator)	22	1	31	1	-	28	27	24	25	6	57
Informed consumer (others)	1	4	7	3	-	9	6	11	4	-	17

### Folk and taxonomic names

Local naming systems distinguish figs according to shape, size, and taste of leaves and fruit (Table 3). Although no common name exists for all the species in the genus, most of the big Bayan trees and related species in the genus *Ficus* start with the local prefix “nyaung” in Burmese languages. For example, *F. concinna* is “nyaung-thabye”, *F. geniculata* and white fig are called “nyaung-chin”, and the sacred fig is “nyaung-bodhi” or “nyaung-taw”. Sometimes, local people name the plants based on the flavor of the edible parts. For example, sour tastes are “chin” in Burmese languages, so a sour young fig leaf bud is called “nyaung-chin-phoo”. For this reason, *F. geniculata* and white fig share the same name “nyaung-chin”. In Shan language, *F. geniculata* and white fig are described as “phak-hee” meaning “the wild vegetable with sour taste”. In the Pa-O language, the word “cha” means edible; the edible female fig of *F. semicordata* is called “thadut-cha”, the edible *F. geniculata* and white fig share the name “kharone-cha”.

**Table 3:** Details regarding eight fig species and their usefulness as food, medicine, and any related conservation practices for 114 informants from five ethnic groups in Southern Shan State, Myanmar

Botanical name	Growth habit	Primary harvest period	Edible parts	Vernacular names					Voucher number
				Burmese (English translation)	Danu	Intha	Pa-O	Shan	
*F. auriculata* Lour.	Small tree (5-10 m)	February–March	Young leaves	Sin-tha-phan (elephant fig)	Phak-ohn/ Phak-wah	Phak-ohn/ Phak-wah	Phak-ohn/ Phak-wah	Phak-ohn/ Phak-wah	EBF 1804 EBF 1813 EBF 1807
		October–December and July–August	Ripe female fig and young green fig						
*F. concinna* (Miq.) Miq.	Tree (< 10 m)	February–April	Young leaves	Nyaung-thabye, Nyaung-pan	Nyaung- Thabye	-	-	Mike-nyaung	EBF 1805
*F. geniculata* Kurz	Large tree (< 20 m)	February–March	Young leaf buds	Nyaung-chin	Nyaung-chin	Nyaung-chin	Kharone	Phak-hee	EBF 1815 EBF 1812
*F. hispida* L.f.	Small tree (5–10 m)	March–April, the whole year	Young leaves	Kha-aung, Pha-aung	Kha-aung				EBF 1809
		The whole year, October–November	Ripe fig and young green fig						
*F. racemosa* L.	Large tree (< 20 m)	February–March	Young leaves	Tha-phan	Tha-phan	Tha-phan	Phak-de	Mike-lay/ Phak-lay	EBE 1810 EBF 1811
		October–December and July–August	Ripe fig and young green fig						
*F. religiosa* L.	Tree (15–30 m)	February–March	Young leaves	Nyaung-bawdi (sacred fig)	Nyaung-taw	Nyaung-ni	Nyaung-ni	Mike-nyaung/ Phak-nyaung	EBF 1803
*F. semicordata* Buch.-Ham. ex Sm.	Tree (< 10 m)	October–December, June–August	Ripe female figs	Kadut	Kadut	Thadut	Thadut	Thadut	EBF 1802
*F. virens* Aiton	Large tree (< 20 m)	February–March	Young leaf buds	Nyaung-chin	Nyaung-chin	Nyaung-chin	Kharone	Phak-hee	EBF 1801 EBF 1808

The Burmese name for roxburgh fig is “sin-thaphan”, which means “elephant fig” due to its large leaf. Danu, Shan, Intha, and Pa-O share the name “phak-ohn” or “phak-wah” for this species, meaning “big leaf fig tree”, similar to the meaning of “elephant fig” in Burmese language. *F. racemosa* shares the same ethnic name “phak-de” for the Pa-O and Danu. Intha and Shan call it “tha-phan”.

*F. regligiosa* is considered a sacred tree across the study region. The Bamar call it “nyaung-bodhi” meaning “Buddha tree”. The Danu people call it “nyaung-taw”, with the respectful “taw” for worshiping Buddha and elders in the Buddhist community. The Intha and Pa-O call it “nyaung-ni” referring to the red colored young leaves. The Shan show their respect for the sacred fig with the name “phak-nyaung” (Table 2) with “phak” for wild vegetable and the respectful affix “nyaung”.

*F. geniculata* and white fig are considered to be distinct species but were given the same local name and local people could not differentiate between them. *F. oligodon* and *F. hainanensis* were considered to be the same ethnospecies as roxburgh fig across local communities and are also described as synonyms for roxburgh fig according to the “World Flora Online” database [38].

### Use value

**Fig. 2:**
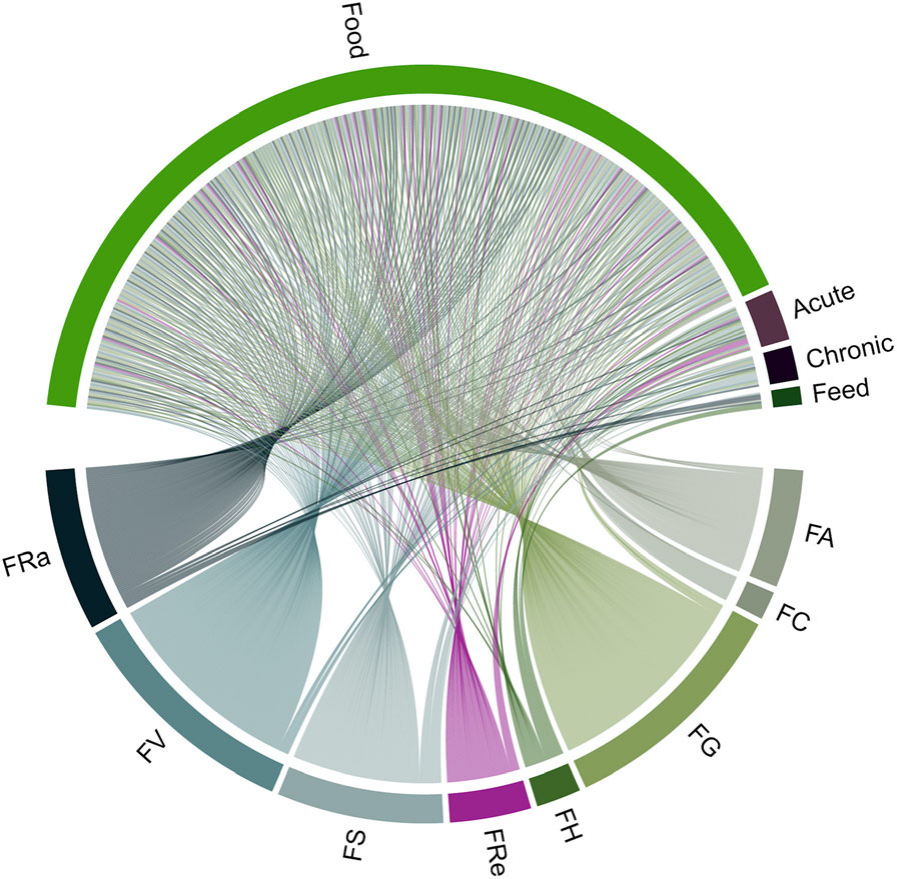
Chord diagram of the distribution of 927 use reports (UR) for eight fig species among 114 informants from five ethnic groups in four townships in Southern Shan State, Myanmar. The diagram shows the four generalized used categories (top half) related to each of the eight fig species (bottom half): **FA** *F. auriculata* Lour. (*F. oligodon* Miq.), **FC** *F. concinna* (Miq.) Miq., **FG** *F. geniculata* Kurz, **FH** *F. hispida* L.f., **FRa** *F. racemosa* L. (*F. glomerata* Roxb.), **FRe** *F. religiosa* L., **FS** *F. semicordata* Buch.-Ham. ex Sm., and **FV** *F. virens* Aivon

There were altogether 927 use reports in all four use categories (Fig. 2). The greatest number of use reports (UR = 228) were recorded for *F. geniculata* and white fig, and each of these species had a correspondingly high use value (UV = 2). These two important species were followed by cluster fig with (UR = 145, UV = 1.27), *F. semicordata* (UR = 114, UV = 1), roxburgh fig (UR = 111, UV = 0.97), the sacred fig (UR = 54, UV = 0.47), *F. hispida* (UR = 29, UV = 0.25), and *F. concinna* (UR = 18, UV = 0.16).

### Generalized used categories

The generalized use categories of figs are shown in Fig. 2. All the eight fig species were mainly used for food followed by acute disease, chronic disease, and animal feed. *F. concinna* was only reported for food. While *F. religiosa* was reported for food and acute disease uses, *F. geniculata* and *F. virens* were reported for food, acute and chronic disease uses, and the rest of the species were reported for all four use categories.

### Food uses

**Fig. 3:**
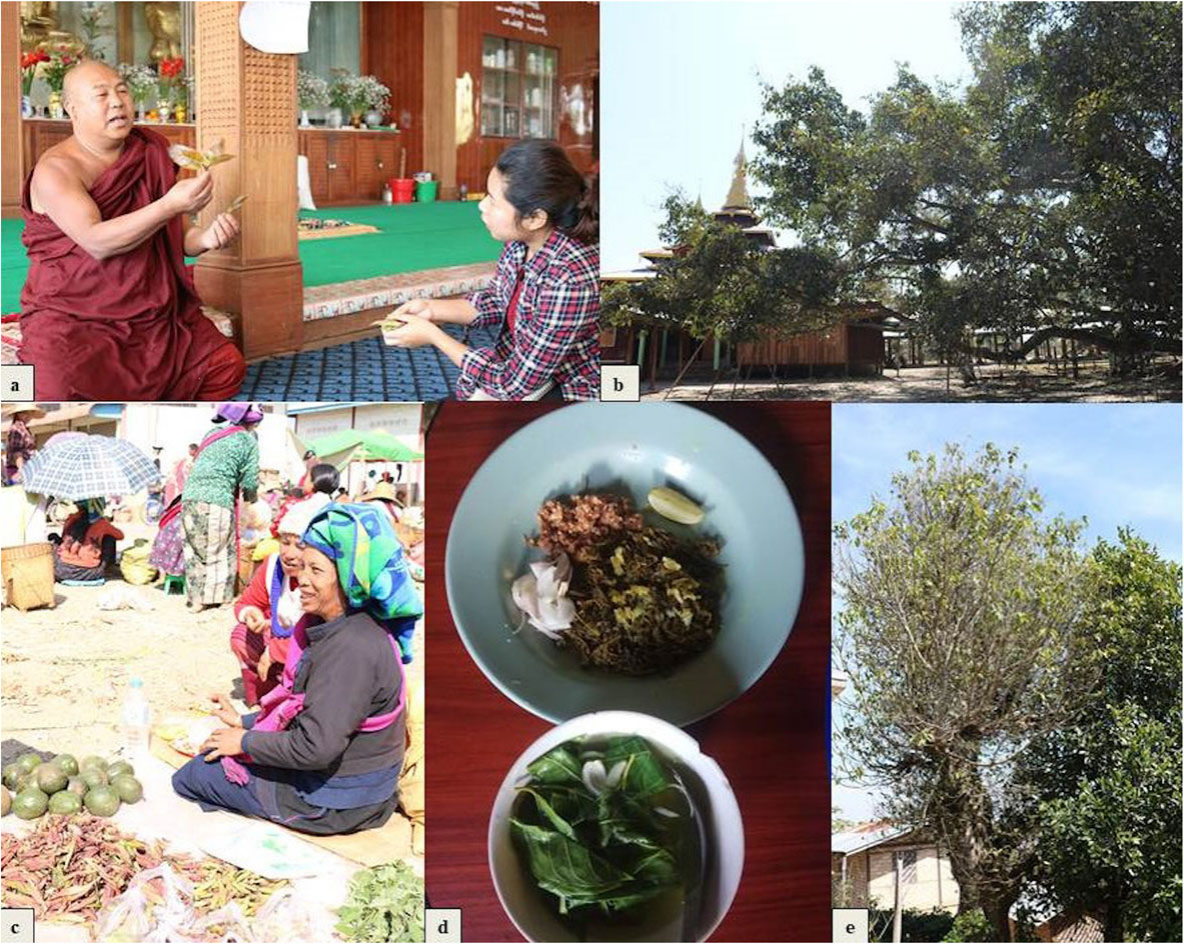
Uses and conservation practices of fig species in Southern Shan State, Myanmar. **a** Monk healer explaining how to use the young leaf of *F. virens* for medicine. **b** *F. religiosa* tree conserving near the Buddha temple in monastery compound. **c** Pa-O women selling the young leaf buds of *F. virens* in local market. **d** a young leaf soup of *F. racemosa* and a young leaf salad of *F. concinna*. **e** *F. geniculata* tree in the local home compound, kept for seasonal food and shade

Most of the uses reported were for food (sum UR *food* = 867) in all four townships (Fig. 3). The young green figs of roxburgh fig, cluster fig, *F. hispida*, and *F. semicordata* were commonly eaten as salad or as a side dish with fish paste and preserved in salt water. The ripe figs of these species are also eaten directly or after making jam with jaggery and sugar as a snack. A homemade beverage is made from the ripe figs of *F. semicordata* by preserving them with sugar for one or more weeks. Only the ripe female figs of dioecious figs are edible, the male fruits are low in nutrients and not palatable [12]. A proverb among Intha people compares the male fruit of dioecious fig with insincere people “although they may look good, there are wasps inside and the taste is poor”. The young leaves or leaf buds of all the reported figs except *F. semicordata* were eaten as vegetables in salad, soup, or fried with rice powder.

Young leaves and leaf buds were the most common edible parts when compared with fruit. They were used more than fruits (sum UR *Young leaves and leaf buds* = 701) and most commonly prepared in soup with peas and beans or in potato soup by all the five ethnic groups. The Intha “saykha-hin” soup is mainly made of cluster fig leaves. Young boiled fig leaves are often paired with tomato sauce or fish paste as a side dish. The mixture of young leaves and rice powder are fried and eaten as a vegetable “pakora”. The young leaves or leaf buds are also preserved with salt in bamboo tubes to be eaten year-round. The pickled young leaves are used in salads and side dishes or as a snack with evening tea. The fruits of *F. concinnna*, *F. geniculata*, white fig, and leaves of *F. semicordata* were not reported to be eaten. The food uses of the young leaf of some fig species are shown in Fig. 3.

### Feeding animals

Fig fruits were used as animal feed and fodder by five informants of Pa-O people and one Danu informant (sum UR *animal feed* = 9). The ripe fig of roxburgh fig was used to feed pigs and *F. hispida*, cluster fig tree, and *F. semicordata* were used to feed both pigs and cattle. Fig leaves were not reported to be used for feed.

### Medicinal uses

**Fig. 4:**
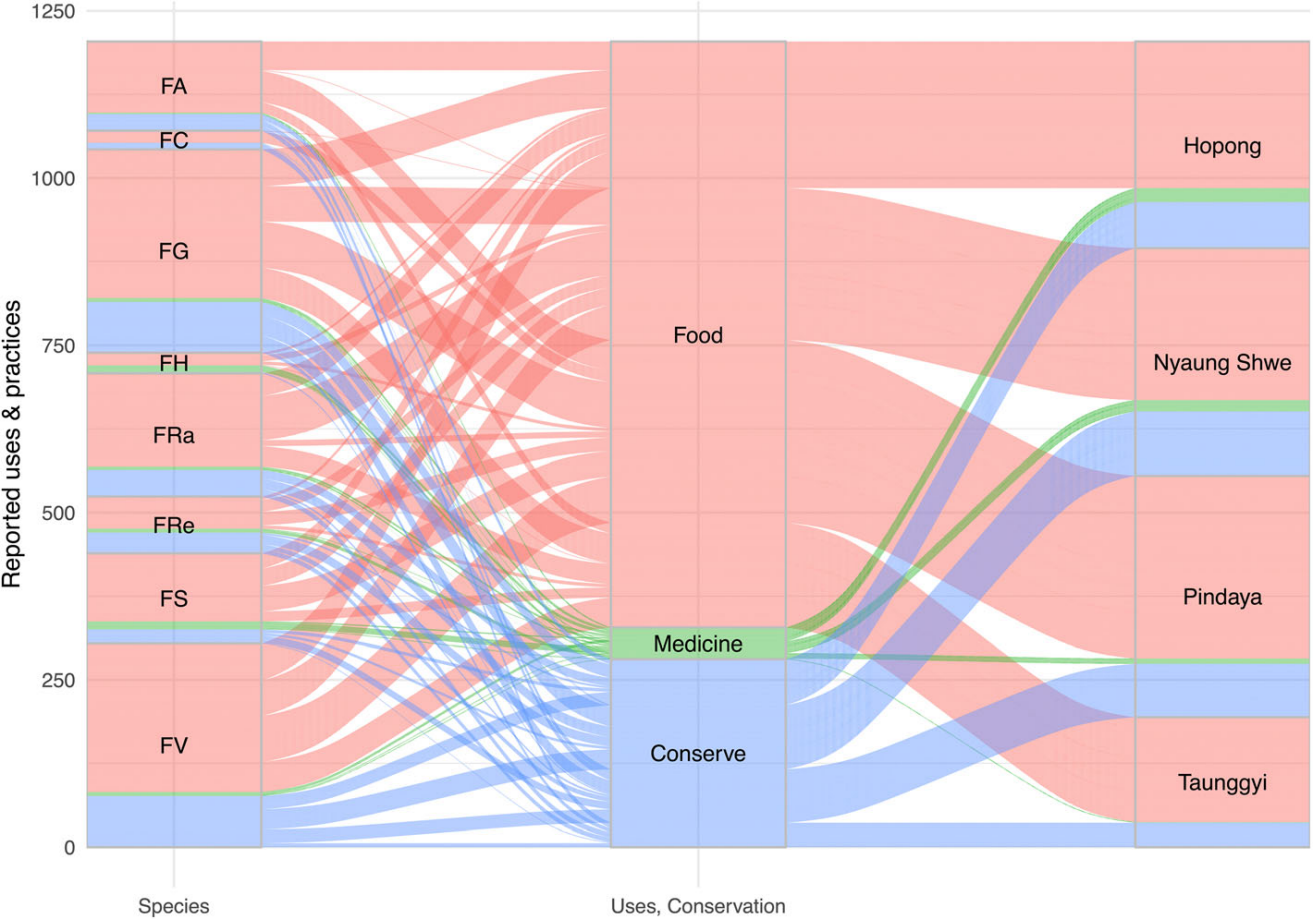
Alluvial plot of the 927 use reports (UR) for eight fig species and their usefulness as food, medicine, and related conservation practices reported by 114 informants from five ethnic groups in four townships in Southern Shan State, Myanmar: **FA** *F. auriculata* Lour. (*F. oligodon* Miq.), **FC** *F. concinna* (Miq.) Miq., **FG** *F. geniculata* Kurz, **FH** *F. hispida* L.f., **FRa** *F. racemosa* L. (*F. glomerata* Roxb.), **FRe** *F. religiosa* L., **FS** *F. semicordata* Buch.-Ham. ex Sm. (*F. cunia* Buch.-Ham. ex Roxb.), **FV** *F. virens* Aivon

Except *F. concinna*, all species are used for medicine across the four townships. They are used for treating 16 major health conditions (sum UR *medicinal uses* = 51). The leaves, fruits, and latex are used to treat topical and internal diseases including poisoning from snake bites (9 UR), the white latex of *F. hispida*, cluster fig, and *F. semicordata* are applied topically; heart disease (6 UR), the ripe fruits of roxburgh fig, cluster fig, and *F. semicordata* are eaten directly or in jam mixed with jaggery or sugar; indigestion (3 UR), the ripe fig of *F. semicordata* is eaten directly, the young leaves of roxburgh fig are eaten as salad; hemorrhoid (1 UR), the decoction of the bark of roxburgh fig is taken orally; ulcers (1UR), the decoction of stem bark juice is applied to the ulcer; cuts and wounds (6 UR), the white latex of the sacred fig is applied directly, the crushed fruit of *F. hispida* is used as a plaster; leucorrhoea (vaginal discharge) (1 UR), the decoction of the bark and leaf of roxburgh fig is taken orally; and urinary diseases (5 UR), the ripe fruit of *F. semicordata* is eaten directly, leaves from *F. geniculata* and white fig are eaten as a soup. This soup and the boiled leaf of cluster fig are also used for treating diabetes (4 UR) and excessive sweating (2 UR) and as a tonic for postpartum health (2 UR). Other medical treatments include diarrhea (4 UR), young leaves of *F. geniculata*, *F. hispida*, and white fig are eaten as side dish after boiling in hot water; constipation (1 UR), ripe fruit of *F. semicordata* eaten directly; fever (1 UR), ripe fig of cluster fig is roasted over fire and taken with salt; hypertension (1 UR), young leaves of *F. hispida* are eaten as side dish after boiling in hot water; irregular mensuration (1 UR), ripe fruit of *F. semicordata* eaten directly or in jam mixed with jaggery or sugar; longevity (1 UR), ripe fruit of *F. semicordata* is preserved with honey or sugar and taken year-round for longevity of elderly patients and those in menopause; herpes (1 UR), latex of cluster fig is applied on the skin; and pulmonary diseases (i.e., asthma and other overabundance of mucus) (1 UR), the ripe fruit of *F. semicordata* is eaten directly. The medicinal uses of all fig species are shown in Fig. 4.

### Economic uses

Economic uses were common (sum UR *economic uses* = 88). The mature leaves of *F. semicordata* were reported to be used for polishing wood by one Danu informant. The young leaves and buds of *F. geniculata*, cluster fig, and white fig were sold as vegetable in local markets in all four townships (Fig. 3). Many of these were found in Hopong market and were reported to be in high demand during their short available season. *F. geniculata* and white fig were sold as one species based on the sizes of leaves and buds. It was estimated that fig sales generate up to 10% of household incomes for fig wild collectors, and up to 5% of household incomes of vendors (vegetable sellers). This income comes primarily during the intensive collection period.

### Conservation status

In general, fig species are well managed and conserved mainly under the influence of religious belief. Conservation practices were reported for all fig species. Informants in all the cultural groups in four townships except Bamar stated that all the species are conserved in the wild as a part of wild collection, especially species in high demand such as *F. genicula* and *F. virens* (111 out of 114 informants reported to use). Communities retain and protect the wild edible figs growing around the villages and farms for seasonal food, shading, and fencing (Fig. 3). Thirty-six (32%) informants reported that they cultivate fig trees in their home compounds for household consumption and that they sometimes sell the surplus.

Depending on the size, age, and location of the individual tree, local people believe that they are home to tree gods. They customarily preserve the tree from as part of the religious custom, and disturbing or damaging the tree is considered taboo. Many of the communities worship a tree god (the guardian spirit of tree) called Yokka-soe who is guarding the sacred fig tree (*F. religiosa*), bayan trees (*F. benghalensis* L.), as well as long-living big trees. People believe that the tree god (Yokka-soe) is benevolent to humans. On the other hand, he may harm a human who misbehaves to him or the tree he is guarding. Fifteen (13%) informants reported that they have their own experience with that when they tried to collect some of the fig trees. While 94 (82%) informants said that they have heard about the taboo from the older generations and the other people who have experienced breaking the taboo. There are five fig species reported to be conserved under traditional taboo because of their size, age, and location in this study—*F. religiosa* (10 reports), *F. racemosa* (3 reports), *F. geniculata* (1 report), *F. concinna* (1 report), and *F. virens* (1 report).

However, there are some actions of local people that have a negative impact on the sustainable use of figs. Firewood collection and small-scale charcoal burning (only reported in Pindaya Township) have minor negative impacts. Local people collect firewood for drying tea leaves. *F. semicordata* is the most cited species for firewood (UR *Firewood* = 78). Some other minor risks are over collection of young leaves and leaf buds for both household consumption and selling. Farmland extensions and road extensions are also threats.

When asked to assess changes in the availability of figs in the past 10 years, 73% of the informants said there were no obvious changes. When asked about the mechanisms contributing to the stability of the tree populations, ninety-four out of 114 informants cited local conservation practices that inform and enforce sustainable collection. The importance of conservation practices across eight species in four townships was summarized based on the reports of 114 informants (Fig. 4).

### Harvesting practice of fig by local ethnic groups

Informants responded that all the eight figs were harvested from the wild. *F. geniculata* and *F. virens* were commonly harvested followed by cluster fig, *F. semicordata* and *F. concinna*, roxburgh fig, and *F. hispida*. According to the informants, the best season for harvesting leaves and buds is in the late winter to summer, and the best time to collect fruits is in the monsoon season. The production of young leaves and leaf buds is supported through pruning. Household consumption is the main purpose for collection, but some surpluses are sold as a source of side income. The local people mentioned that fruits were mainly collected by children as a snack, but they were not often eaten by adults and are left to grow wild. However, figs that are a source of fruit (e.g., roxburgh fig, *F. hispida*, and *F. semicordata*) are harvested twice annually, during peak fruit production from June to August and from October to December but can be collected in lower abundance throughout the year. The informants reported that the harvest times can vary according to weather and geographic conditions.

### Comparative uses among ethnic groups

**Fig. 5:**
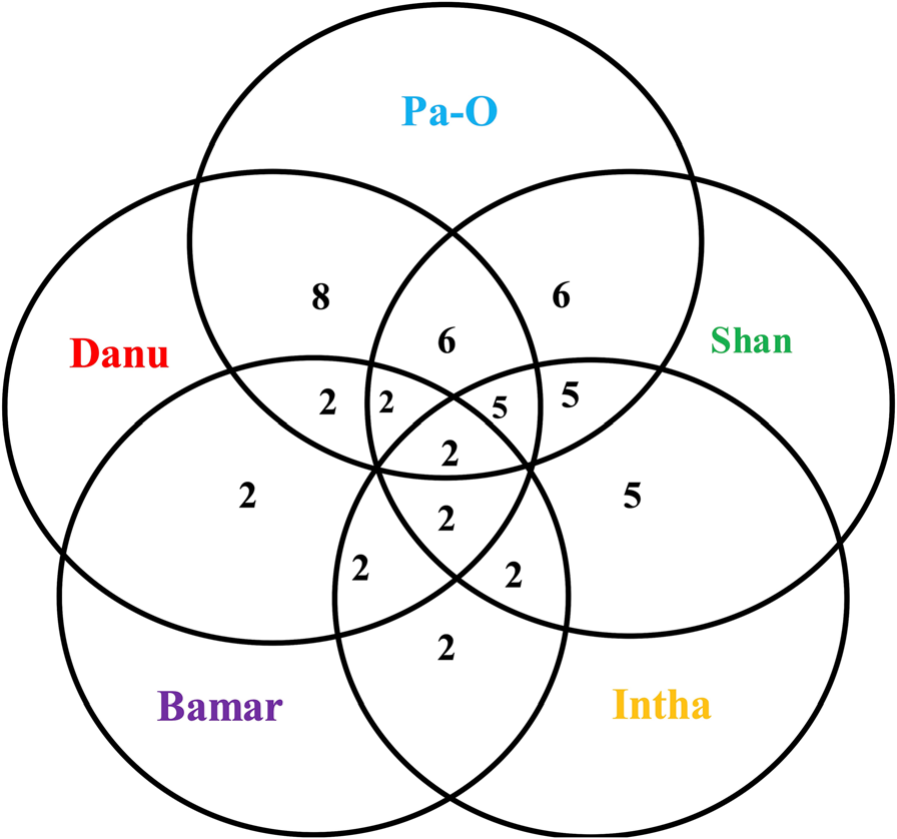
Venn diagram showing the overlap in uses for eight fig species among 114 informants from five ethnic groups in four townships in Southern Shan State, Myanmar. The overlap between circles represents the number of species that are commonly used between ethnic groups. No species were unique to a specific ethnic group

The number of species used was different among ethnic groups and geographic locations (Fig. 5). Danu and Pa-O had the greatest number of uses followed Shan, Intha, and Bamar. The Danu and Pa-O shared uses for eight figs. Danu, Pa-O, and Shan share uses for six species (roxburgh fig*, F. geniculata*, cluster fig, the sacred fig, *F. semicordata*, and white fig*.*). The Intha ethnic group shares five species with Danu, Shan, and Pa-O (*F. geniculata*, cluster fig, the sacred fig, white fig, and *F. semicordata*). The Bumar are the smallest informant group (*n* = 4) and only reported two figs (*F. geniculata* and white fig), which were also common to all other ethnic groups in the study area.

## Discussion

### Fig species identification

For the eight fig species in our study, our approach of species identification using photos and samples was an effective tool. Based on these, informants were also able to indicate additional characteristics related to fruit, latex, young leaves or leaf buds, leaf sheath, and stipules not shown in the photos and specimens. This helped us with identifying materials in the market. In the case of *F. geniculata* and white fig, they were considered to be the same ethnospecies but are different species according to botanical taxonomic identification. Similar approaches may be useful for other studies to differentiate between different species.

### Folk names and species confirmation

Gathering local names and learning about naming conventions was also useful in defining monophyletic groups of fig species. Roxburgh fig, *F. oligodon*, and *F. hainanensis* were also the same ethnospecies among Danu, Pa-O, and Shan. Molecular phylogeny research studies have revealed the same three forms as one species [45], and the three were once considered synonyms of roxburgh fig [38]. It may be worthwhile to study more of the similarities among local names and molecular phylogeny. In this case, using molecular analyses such as sequence analysis of highly conserved genes and intergenic spacers of chloroplast DNA could provide a clearer picture of differentiation between species.

### Food uses of fig species compared to other studies

The use of fresh fruits of cluster fig and *F. semicordata* were also reported elsewhere in Southern Shan State [19]. The frequent use of *F. auriculata*, *F. hispida, F. racemosa, F. religiosa, F. semicordata*, and *F. virens* among others, is also similar in neighboring countries such as Vietnam, India, China, Nepal, and Thailand [12, 46–49]. The young shoots and young leaves of *F. geniculata* and *F. virens* are also considered edible in Thailand [48]. According to [50, 51], the fruits of *F. hispida* are poisonous and can cause intestinal irritation which may lead to death when eaten. However, the ficin (the proteolytic enzyme included in the latex) is an effective cure for intestinal worms such as roundworms and hookworms [52]. Interestingly, no informant in our study, not even local healers, reported that the fruit is harmful. Similar findings on edible leaf shoots and fruits of *F. hispida* has been reported in Malaysia [53]. Although no toxicity information was reported in the current study, this species should be studied further to determine the efficacy and safety evaluation for consumption.

### Feeding animal

Another interesting finding from our study was that only the fruits of four figs—roxburgh fig, *F. hispida*, cluster fig, and *F. semicordata*—were used as animal feed. Figs are commonly used as animal fodder in the Himalaya region and India especially for roxburgh fig [46, 54–56]. The difference may be due to the year-round availability of fruits compared with young leaves, which can be collected only during a short season.

### Medicinal importance

Monks are locally considered to be the source of wisdom [57]. They remain a primary source of health care. In matters of health all the ethnic groups except a few Bamar in this study rely on traditional plant medicines and consult local practitioners to cure their ailments. In the study area, seven out of eight edible figs had some medicinal use. In some cases, diet and medicine are difficult to distinguish [58], i.e., when preparing the leaves for healing soup or eating the ripe fruit specifically for medicinal benefits. Other medicinal uses were more obvious and easier to assess, i.e., topical treatments of latex and crushed fruit. Other studies have cited similar medicinal uses for these figs. In Nepal, the tender shoots and fruits of roxburgh fig are used to treat diarrhea and dysentery [59], and stem bark juice is used to treat cuts and wounds [46]; the ripe fruit of *F. semicordata* is eaten to treat constipation and indigestion [59, 60]; the raw fruit is used to cure diarrhea [61]; and the white latex is used to treat boils [60]. In Pakistan, the leaves of the white fig, decoction of the fruits of the cluster fig, and the infusion of bark and decoction of the fruit of *F. hispida* are used to treat diabetes [62, 63]; the ripe fruits of *F. semicordata* are taken for diarrhea, and the ripe fruits of the cluster fig are taken for heart disease [63]. In north Pakistan, the powder from unripe fruits of the cluster fig are eaten for intestinal worms, piles, and menstrual disorders [64]. In Nepal, the white latex of the same tree is used to treat muscular pain, cut and wounds, fractures, and boils, and the infusion of the leaf is used in menorrhea [46]. In India, the leaf juice of sacred fig is consumed to treat asthma, cough, diarrhea, and gastric problems [8], while the leaf juice and honey are taken for diarrhea [65], and the stem bark is used to cure cuts and wounds [66] in Nepal. Medicinal figs should be further studied for their role in local health care, especially for those rural communities with limited access to conventional medicine.

### Socioeconomic importance

Our findings indicate that figs were also of socioeconomic importance for rural people. We found diverse markets with some figs such as *F. racemosa*, *F. geniculata*, and *F. virens* sold as vegetables. This is consistent with other studies on the marketable importance of figs for food. Young fruit and leaves of *F. auriculata*, the young leaves of *F. oligodon*, and the leaf buds of *F. virens* are also sold on the local markets of Yunnan, China [12]; the near-ripe peeled or unpeeled fruits and young leaves of *F. auriculata* were sold in the markets of north and central Vietnam [47]; and the fruits of *F. auriculata* were sold in the cities in north Karnataka [67]. Moreover, the mature leaves of *F. semicordata* were used for polishing wood in India [68]. Over exploitation of marketable species from the wild may threaten the future availability. If demand grows, there may be a need for widespread promotion and awareness-raising of traditional sustainable wild collection practices.

### Conservation status

The availability of wild plant resources for both subsistence and markets depends upon sustainable harvests, appropriate management, and the domestication of wild resources [69]. In this study, we observed cultural and indigenous knowledge-related conservation practices for figs, passed down across generations. The villages in all the cultural groups in four townships except Bamar take action toward *in situ* conservation practices, as well as domestication of edible figs. Promotion of these local customs related to sacred fig may result in better conservation strategies. The taboo keeping people from cutting fig trees is strong since many believe that it can be harmful to break taboo [70]. Based on this custom, the sacred figs are protected from cutting or overharvesting under the supervision of monks, especially those trees that are grown in the monastery compound. The culture of conserving figs for sacred and religious purposes is common not only in Myanmar but also in other parts of the world [7]. Even though fig trees are reportedly used for firewood, local people mentioned that figs are not really a good choice for firewood but can be used only after drying and is only used when there is no other choice. The risk of over collection is negligible when compared with the positive practice of *in situ* and *ex situ* conservation. *In situ* conservation management can help to maintain natural populations of fig species in their natural habitats. The success of such management practices depends on rules, regulations, and compliance [71]. Related interventions can support the conservation of figs. For example, a grant from the Rufford Small Grants Foundation from the UK promoted cultivation and conservation of two figs (1) *F. virens* and (2) *Ficus altissima* with the participation of the local nature conservation committee, regional women’s and welfare associations, and volunteers in 116 villages of Pindaya Township in 2008 [72]. They introduced awareness-raising on the importance and the ecological role of fig trees to the relevance local groups including school children. More of such awareness-raising initiatives for sustainable utilization of figs should be performed with local communities in cooperation with national and international organizations to promote traditional knowledge on the sustainable use of natural resources. Further studies on the potential for expansion of existing traditional conservation practices may be part of the solution for maintaining the forest ecosystems of Myanmar.

## Conclusions

Our study illustrates the importance of figs for local diets and medicine and in supporting side incomes and feeding domestic animals across ethnic groups in Myanmar. The local ethnobotanical knowledge on the use of these figs has been transmitted orally and handed down through many generations of citizens, whereas medicinal knowledge is the realm of monks and healers. Moreover, traditional local customs and taboos regarding *in situ* and *ex situ* conservation practices were important for sustainable utilization in the area. Harvesting and other extractive uses such as collecting firewood are unlikely to have a negative effect on figs. However, extension of farmlands and roadways may put serious pressure on availability of figs species in the future. The conservation of figs and the preservation of traditional practices should be part of conservation efforts by local and national organizations. In line with these efforts, more exploration and documentation of ethnobotany knowledge of the ethnic minority groups should be conducted. Further studies should also address the nutrient constituent and antioxidant activity of the edible figs explored in this study and throughout the country. The ethnic groups should be acknowledged as owners of the documented traditional knowledge, valuable to the identity of future generations. The benefits that may arise from the research should seek to contribute the preservation of the flora of Myanmar and support the sustainable development goals of Myanmar.

## Data Availability

The datasets used and analyzed during the current study are available from the corresponding author on reasonable request.
